# Small airway dysfunction and poor asthma control: a dangerous liaison

**DOI:** 10.1186/s12948-021-00147-8

**Published:** 2021-05-29

**Authors:** Marcello Cottini, Anita Licini, Carlo Lombardi, Diego Bagnasco, Pasquale Comberiati, Alvise Berti

**Affiliations:** 1 Allergy and Pneumology Outpatient Clinic, Bergamo, Italy; 2 Departmental Unit of Allergology, Immunology and Pulmonary Diseases, Fondazione Poliambulanza, Brescia, Italy; 3 Allergy and Respiratory Diseases, IRCCS Policlinico San Martino, University of Genoa, Genova, Italy; 4 Section of Pediatrics, Department of Clinical and Experimental Medicine, University of Pisa, Pisa, Italy; 5 Ospedale Santa Chiara and Department of Cellular, Computational and Integrative Biology (CIBIO), University of Trento, Trento, Italy; 6 Thoracic Disease Research Unit, Mayo Clinic, Rochester, MN USA

**Keywords:** Asthma, Small airway diseases, IOS, FOT, Impulse oscillometry, Asthma control, Lung function

## Abstract

Asthma is a common chronic condition, affecting approximately 339 million people worldwide. The main goal of the current asthma treatment guidelines is to achieve clinical control, encompassing both the patient symptoms and limitations and the future risk of adverse asthma outcomes. Despite randomized controlled trials showing that asthma control is an achievable target, a substantial proportion of asthmatics remain poorly controlled in* real life.* The involvement of peripheral small airways has recently gained greater recognition in asthma, and many studies suggest that the persistent inflammation at these sites leads to small airway dysfunction (SAD), strongly contributing to a worse asthma control. Overall, the impulse oscillometry (IOS), introduced in the recent years, seems to be able to sensitively assess small airways, while conventional spirometry does not. Therefore, IOS may be of great help in characterizing SAD and guiding therapy choice. The aim of this article is to review the literature on SAD and its influence on asthma control, emphasizing the most recent evidence.

## Background

Asthma is a common chronic condition in the world and the most common non-communicable disease among children, affecting approximately 339 million people of all ages, races and geographic origins [1, 2]. It is estimated that over 100 million more people will be affected by 2025 [1, 2]. In Europe, asthma affects 30 million people and is associated with a significant socioeconomic burden [3], representing the 14th most important disorder in terms of global years lived with disability, according to the Global Burden of Disease Study data [2].

One of the main long-term goals of asthma management is to achieve a good disease control, by repeatedly reviewing patient’s symptoms (daytime symptoms, nocturnal symptoms, activity limitations and use of rescue medications) and future risk of exacerbation, adverse effect of therapy and lung function decline, and by adjusting the treatment accordingly [4]. Current recommendations are therefore based on the level of asthma control rather than disease severity [4]. Despite randomized controlled trials showed that asthma control is an achievable target [5, 6] real-life studies in the last 20 years have shown that a substantial proportion of asthmatics remain under-controlled [7–17], even in those patients receiving treatment from an asthma specialist [18, 19] and in those with mild asthma regularly treated with inhaled corticosteroids (ICS) [20] Ultimately, asthma control has been shown to be sub-optimal across all GINA steps [21].

Poor asthma control is associated with increased risk of exacerbations, impaired quality of life, increased health-care utilization and reduced productivity [22, 23]. History of asthma exacerbations, poor treatment adherence, failure to use inhalers correctly, heterogeneity of asthma phenotypes and associated comorbidities have been shown to be the main contributors to poor disease control [24–32]. More recently, the persistence of uncontrolled inflammation in the peripheral small airways emerged as a strong contributors of asthma control [33–36].

Herein we reviewed the evidence supporting the influence of small airway dysfunction (SAD) on asthma control, emphasizing the most recent one, and highlighting how the identification of SAD by techniques different than conventional spirometry may be useful to assess SAD, potentially guiding asthma treatment.

### Small airways dysfunction (SAD)

Even if asthma affects the entire bronchial tree [37], small airways has been recognized as the major site of airflow limitation in both asthma and chronic obstructive pulmonary disease [38, 39].

The small airways are defined as airways with an internal diameter ≤ 2 mm that do not contain cartilage in their walls and extend from the 8th generation airways to the periphery of the lung [40]. Under normal circumstances, small airways contribute only minimally to airway resistance, and for this reason they are known as the lung’s “quiet zone” [41]. In contrast, in COPD and asthma, small airways are likely the key area that determines the transition from physiologic to pathophysiologic behavior of the bronchial tree [38]. Many studies and systematic reviews suggested that SAD is associated with more severe bronchial hyper-responsiveness, worse asthma control and a higher number of exacerbations [33–36, 42].

Overall, the prevalence of SAD in patients with asthma is around 50–60% [43], but it seems to vary with the physiological measure used to assess it [33]. The In the multinational study ATLANTIS [33], the largest study to date dealing with the contribution of SAD to asthma severity, SAD was strongly present across all GINA severity stages. Even if the prevalence changes is considerably depending on the physiological variable used to assess SAD, it remains consistently higher in more severe asthma (GINA step 5) [33]. We contributed by showing that the true prevalence of SAD measured by impulse oscillometry (IOS) in a cohort of 400 community-managed patients with physician-diagnosed asthma was 61.5%, and SAD was present in the majority of subjects across all the classes (step 2 58.3%; step 3 60.9%; step 4 63.3%; step 5 78.6%; p > 0.05) (Cottini M et al., Respiratory Medicine, submitted).

### Small airways assessment

According to the current Global Initiative for Asthma (GINA) guidelines, spirometry remains the method of choice in evaluating the respiratory function [25]. However, conventional spirometry reflects mostly the variability and/or the reversibility of airway obstruction and is unable to sensitively evaluate small airways, becoming abnormal on spirometry only when approximately 75% of small airways are obstructed [44–46]. Therefore, small airways are difficult to access, and the lack of standardized and unanimously accepted methods of measurement has often left the assessment of the small airways to the experimental and investigational level. In the recent years more specialized tests have been developed, moving from clinical research laboratories into routine clinical practice [47, 48]. Table 1 summarizes the techniques available for the assessment of small airways disease. No assessment method is universally and directly representative of peripheral airway function [42, 47, 48].

**Table 1:** Available techniques for the assessment of bronchial airways by size (small versus large airways)

Method	Small airway function	Large airway function
Spirometry	FEF25–75%, FVC, FVC/SVC	FEV1, FEV1/FVC
Impulse oscillometry (IOS)	R5–R20, X5, AX, Fres	R20
Single breath nitrogen washout (SBNW) or Multiple breath nitrogen washout (MBNW) test	Slope phase III, CV, CC, Sacin, Scond	
Body plethysmography	RV, RV/TLC	
High resolution computerized tomography (HRCT)	Air trapping, airway wall thickness	Airway wall thickness
Nuclear medicine (Scintigrapy, SPECT, PET)	Regional ventilation defects	
3He-MRI	Non-ventilated lung volume	
Bronchoscopy	Transbronchial biopsy, BAL	Endobronchial biopsy
Sputum induction	Late phase sputum	Early phase sputum
Exhaled nitric oxide (eNO)	Alveolar eNO	Bronchial eNO
CT and computational fluid dynamics	Changes in airway volume and resistance	

#### Conventional spirometry

The correlation between conventional lung function measurement (FEV1, PEF) and asthma symptoms is weak [4, 49, 50]. This may be due to an airflow dysfunction in the small airways that is not reflected in the FEV1 responses [51]. The mean forced expiratory flow (FEF) between 25 and 75% of FVC (FEF25–75) is the traditional index of spirometry to assess peripheral airways obstruction in routine clinical practice [48, 52].

Some studies suggest that FEF25–75 associates with worse asthma control and poor asthma outcomes. Siroux et al. showed that small-airway obstruction, as assessed based on FEF25–75, might contribute to the long-term persistence of asthma and the subsequent risk for poor asthma outcomes independently from effects of the large airways [53]. Riley et al. [54] showed that FEV1, FEV1/FVC, and a reduced FEF25–75% was independently associated features of more severe asthma, in patients with severe disease.

Despite this, the value of FEF25–75% as a predictor of peripheral obstruction has also been questioned by several studies [55, 56], therefore limiting its reliability for SAD.

#### Impulse oscillometry

The forced oscillation technique (FOT) was first described by DuBois in 1957 as a method to characterize respiratory impedance [57]. The device generates sinusoidal sound waves that are transmitted into the respiratory system during quiet breathing. The modified method, impulse oscillometry (IOS), was developed by Michaelson in 1976 [58] and commercialized by Jaeger in the 90s. IOS operates by delivering a continuous spectrum of frequencies [59–62]. Similar to FOT, the IOS technique uses pressure pulses delivered into the respiratory system, causing a flow reaction, but pressure oscillations in IOS in contrast to FOT are delivered to the respiratory system at a constant frequency (square waves) of 5 Hz, from which all other frequencies of interest are mathematically extracted [59, 63].

IOS is a simple and noninvasive method, requiring minimal patient cooperation, without the need for a shutter, body plethysmography cabin, or measurement gases. Patients can comply better with tidal breathing, compared to maximal inspiratory and expiratory maneuvers, allowing measurements in patients groups who would struggle using conventional methods, requiring forced expiratory manoeuvres that can be difficult or sometimes even impossible to perform. These include children under the age of 5, obese, geriatric patients, patients with limitations of their respiratory drive, severe diseased and patients with neuromuscular abnormalities. For instance, in a study comparing oscillometry and spirometry in patients 65 and older, all were capable of producing a valid oscillometry test whereas valid spirometry was completed in only 33.4% of the participants [64].

The European Respiratory Society (ERS) recently published technical standards for oscillometry measurement in the European Respiratory Journal [65, 66].

**Fig. 1:**
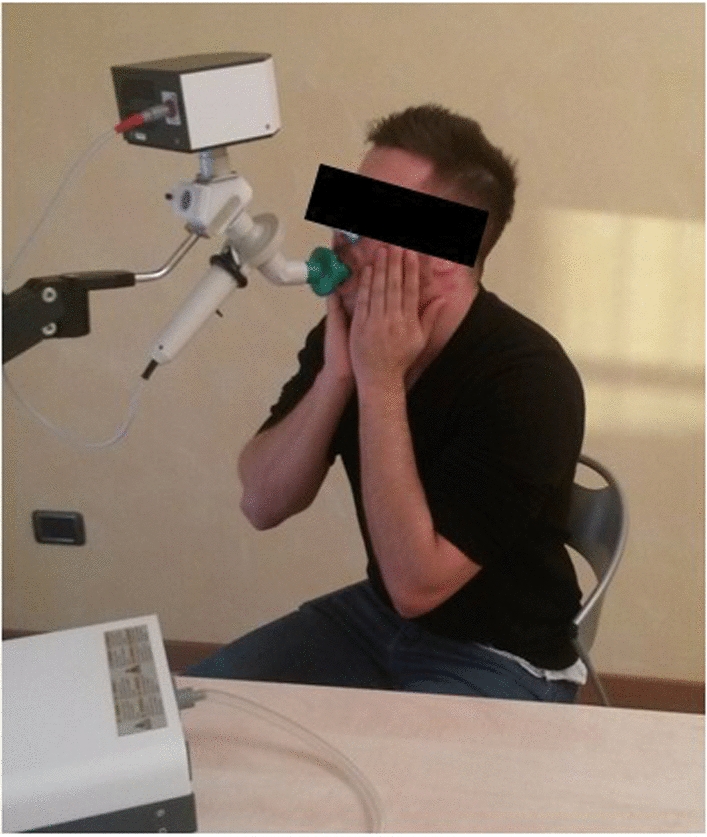
Explanatory figure of a patient tested by impulse oscillometry

In our Allergy and Pneumology Outpatient Clinic, IOS (Masterscreen IOS/Sentry Suite, VyAire Medical) is performed in triplicate in accordance with manufacturer’s and European Respiratory Society guidelines [65, 67]. The IOS system is routinely calibrated, as suggested by the manufacturer [67]. Patients were asked to wear a nose clip and were seated during tidal breathing with their neck slightly extended and their lips sealed tightly around the mouthpiece, while firmly supporting their cheeks with their hands (Fig. 1).

**Fig. 2:**
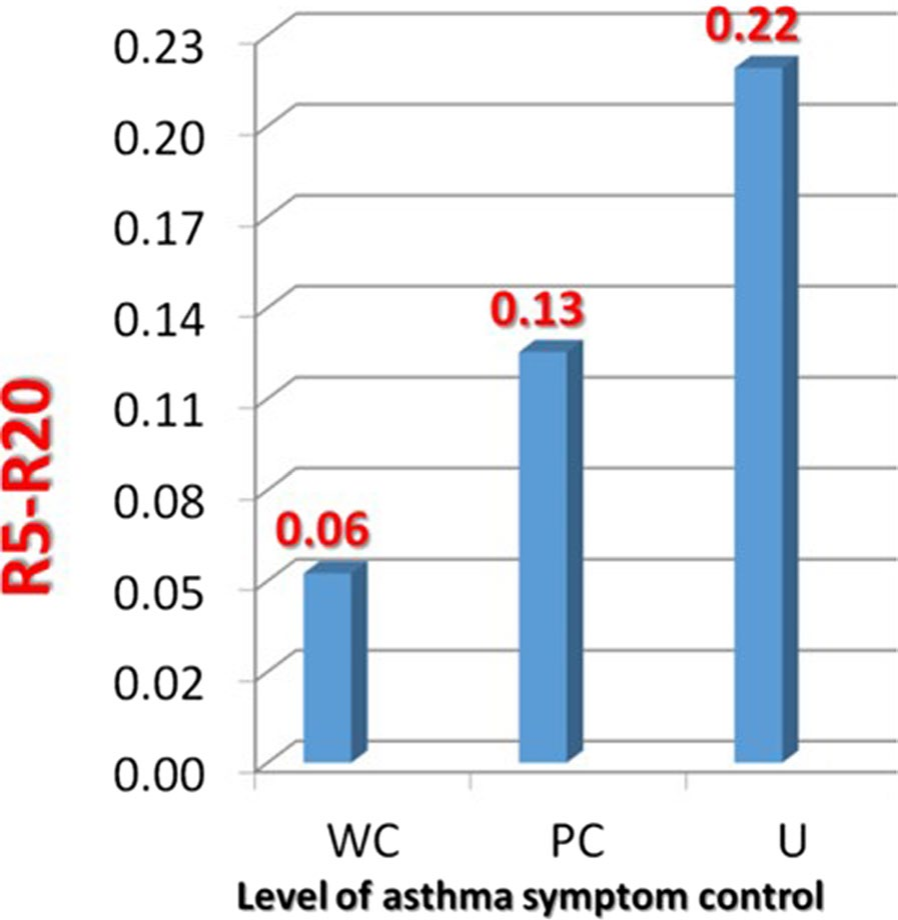
Relationship between R5–R20 measurements and GINA control categories (from [34])

Assuming the coherence, which is a measure of testing reliability, is acceptable (> 0.80 at 10 Hz) and if no evidence of coughing, swallowing, vocalization, or breath holding, the trial is saved. At least three trials are performed, each lasting 30 s, and mean values are chosen (Fig. 2).

The clinical interpretation of the measurements is usually based on the two components of respiratory impedance Zrs, respiratory resistance Rrs and lung reactance Xrs. Both Rrs and Xrs, which reflect total pulmonary impedance, are measured by the investigator in real time as a function of flow volume and pressure. Respiratory resistances at 5 and 20 Hz (R5 and R20, in kPa × s × L^−1^) are used as indices of total and proximal airway resistance, respectively. Thus, the contribution of the distal airways are determined by the fall in resistance from 5 to 20 Hz (R5–R20, in kPa × s × L^−1^), that is considered to be an index for the resistance of peripheral airways, as already performed in asthmatic patients in clinical trials and hospital cohorts, and an R5–R20 cutoff of > 0.07 kPa × s × L^−1^ (a conservative upper limit of normal for R5–R20 as previously reported) is conventionally chosen to define the presence of SAD [34, 61, 62, 68, 69]. Moreover, reactance at 5 Hz (X5, in kPa × s × L^−1^), reflecting elastic recoil of the peripheral airways, resonant frequency (Fres, in Hz), defined as the frequency at which the inertial properties of airway and the capacitance of lung periphery are equal, and reactance area (AX, the area under the reactance curve, in kPa/L), reflecting the elastic properties of the lung periphery and shown to be correlated with resistance at lower frequencies, are also collected [50, 51, 55].

The respiratory resistance Rrs measured by oscillometry differs slightly from the airways resistance measured using body plethysmography (Raw) and the resistance acquired by interrupter technique (Rint, Rocc), which is due to differences in the measurement principles [63].

Thanks to its ability to differentiate between central and peripheral abnormalities, IOS supports individualized patient management, independent of other functional examinations or as part of additional diagnostic measurements [51, 52, 55]. Several studies showed the usefulness of IOS to detect SAD: the ATALANTIS study stratify SAD into two clinically meaningful groups by use of IOS and spirometry [33]. Interestingly, this study identified R5–R20 as the IOS-measured marker that, among several small-airway physiological markers, most strongly correlated with SAD [33]. Other studies showed that R5–R20 reflects small airway narrowing [70, 71]. Another group showed that small-airway ventilation heterogeneity captured by IOS-derived R5–R20 values is associated with CT density gradient reversal at the lung base, likely a direct consequence of SAD [72].

Finally, we recently showed that R5–R20 closely correlates with that of X5, AX and Fres, representative markers of peripheral airway abnormalities, supporting R5–R20 as a surrogate of peripheral airway mechanics [34].

IOS has been shown to detect expiratory flow limitation (EFL), a phenomenon which occurs when increased expiratory effort and driving pressure do not result in increased corresponding flow, due to regional “choke points” from airway closure or narrowing in the distal airways. EFL can occur in asthma and is a pathological hallmark in COPD, often manifesting as dynamic hyperinflation and increased exertional dyspnoea resulting in exercise limitation [47].

For all these reasons, measuring IOS in a *real-life* setting should complement spirometry as part of the routine work-up of asthma patients. However, the method does have some limitations: first, there are no uniform standards to facilitate a set of unified results and the development of a standardized method for calculating values based on the measured parameters [55], but there is now clear guidance about the approach to calibrating oscillometric systems and the way in which testing should be conducted [66]. Our lab uses the manufacturer recommends equations published by Vogel and Smidt in 1994. For adults, they recommend that R ≥ 150% (all frequencies) and R5–20 ≥ 20% be considered abnormal [67]. The rationale and pros and cons for using these values are outlined in a review chapter by Smith and colleagues [50]. Second, IOS/FOT present difficulties when comparing measurements taken by different devices. With several commercially available devices measuring respiratory impedance by oscillometry, the agreement between values obtained on different instruments or frequencies remains unclear. Future studies may have additional value by obtaining normal reference values of IOS measurements and to establish comparability between different instruments.

#### Multiple-breath washout techniques

Many studies assessed ventilation heterogeneity: increasing unevenness of ventilation between different lung regions is a sensitive marker of abnormal small airway function and can be measured noninvasively by using the single-breath washout (SBNT, increase in the phase III slope, dN2) or multiple-breath washout techniques (MBNW) [73, 74]. MBNW is able to distinguish between ventilation heterogeneity generated in the conductive lung zone (Scond) and ventilation heterogeneity generated in the acinar lung zone (Sacin) [45].

Different studies from independent groups showed that ventilation distribution was abnormal in a remarkable proportion of asthmatic patients, of whom only a fraction had an abnormal FEV1 [75], and ventilation alterations were associated with worse asthma control, exacerbations, higher ICS dose [76–82].

#### Imaging: inhaled gas magnetic resonance and computed tomography

Ventilation heterogeneity may be regionally identified using pulmonary imaging methods including inhaled gas magnetic resonance imaging (MRI). In asthma patients, MRI has revealed persistent ventilation heterogeneity, although its relationship to asthma control is not well understood. In patients with poorly controlled, severe asthma MRI ventilation, but not lung clearance index (LCI) was significantly worse in those with worse ACQ and AQLQ [83].

Computed tomography (CT) has emerged as a useful tool to assess peripheral airways disease noninvasively in patients with asthma. The cardinal CT sign of peripheral airways disease in asthma is the presence of pulmonary decreased attenuation areas, which are more consistent on expiratory CT scans diseases [47]. High-resolution CT allows direct assessment of large and medium airways (diameter > 2–2.5 mm), and indirect assessment of small airways. Areas of mosaic lung attenuation on inspiration during CT and air trapping on expiratory CT have been evaluated as markers of small airways disease in both asthma and COPD [47]. Asthmatic patients with air trapping were significantly more likely to have a history of asthma-related hospitalizations, ICU visits, and/or mechanical ventilation [84]. CT does have some limitations, including a lack of standardization of technical parameters for the CT scanner, a lack of consensus on the best index for small airways disease assessment, and exposure of subjects to ionizing radiation.

#### Exhaled NO

Studies in adults and children showed that patients with increased alveolar NO levels more frequently had visits to the emergency department, severe attacks, and hospitalizations [85, 86]. In addition, the alveolar component of exhaled NO is associated with the lack of asthma control in patients with mild, untreated asthma [87], supporting the hypothesis abnormalities of the peripheral airways are involved even in the mildest forms of asthma.

### SAD and asthma control

A growing body of literature correlates IOS with asthma features, and overall, it emerges that **asthma control** appears to be linked with SAD.

In a study in 65 well-characterized patients, IOS-defined SAD correlated better with clinical symptoms and asthma control than spirometry-defined SAD; furthermore, greater small-airways reactance was associated with loss of asthma control [88]. Pisi et al. showed that IOS-defined SAD was associated with poor disease control, assessed by the Asthma Control Test in 33 adult asthmatic patients with normal FEV1 [89]. Manoharan et al. evaluated adult asthmatics with a preserved FEV1 (> 80% predicted), and showed that SAD assessed by FEF_25–75%_, and R5–R20 was associated with a significantly increased likelihood of having worse long-term asthma control [90]. These results were confirmed by other studies with a similar design [91–93].

Notably, the risk of having poorer asthma control was greater when measurements of FEF_25–75%_, and R5–R20 were combined [90]. R5–R20 and AX were closely related to asthma control assessed by asthma control questionnaire (ACQ), while spirometry did not [90]. In ATLANTIS study [33], a SAD score has been calculated by use of impulse oscillometry and spirometry and associated significantly with asthma control, history of exacerbation and disease severity. The highest correlations were seen for airway resistance and reactance (R5, R5–20, reactance at 5 Hz, and reactance area), peripheral conducting airway heterogeneity (Scond), and hyperinflation (residual volume/total lung capacity) [33].

### Quality of life

Asthma control and SAD are lumped together. Kuo et al. showed that peripheral lung resistance and reactance measured by Airwave Oscillometry System-AOS (TremoFlo C-100 Thorasys, Montreal) are related to patient reported outcomes of asthma control and quality of life [94].

Foy et al. demonstrated that IOS-defined SAD has a marked impact on both asthma control and quality of life and may be modified by biologics [95]. Kuo et al. retrospectively demonstrated that IOS-defined SAD was associated with worse asthma control and type 2 inflammation [96].

Other studies showed that FEF_25–75%_ was not as good as IOS to identify SAD [34, 93]. In a cohort of stable asthmatic patients, lower baseline ACT scores correlate with measure of increased baseline peripheral airway dysfunction using IOS, but not with spirometry, supporting the use of objective non-invasive techniques to detect increased airway resistance in a population of stable asthmatic individuals [97].

Interestingly, IOS values are significantly different between uncontrolled, partially controlled, and controlled GINA definition in asthmatic subjects [98]. In our cohort of 400 patients with physician-diagnosed asthma [34], IOS-defined SAD was present in virtually all the patients with uncontrolled asthma, in two third of those with partially controlled asthma, and in one third of those with well-controlled asthma. In parallel, R5–R20 progressively increase from well controlled to uncontrolled asthma, reflecting a more severe SAD with the worsening of control. In general, all IOS measurements, i.e., R5, X5, R5–R20, Ax, Fres, progressively worsened with the aggravation of asthma control categories (p < 0.0001 for all combinations) [34].

Interestingly, in elderly asthmatic patients of our cohort, a true prevalence of SAD of 84.1% compared to 57.3% (*p* < *0.01*) in nonelderly [99]. In elderly patients, SAD is more often associated with central airway disease. Age significantly correlates only with R5–R50, X5, Ax, but not with Type 2 biomarkers and standard spirometry measurements. Older age was strongly associated with worst asthma control [99].

Taken altogether, asthma control therefore is more intimately related to the IOS-defined SAD phenotype than to FEV1 per se and this strongly support the importance of using IOS.

### Predictors of SAD

SAD was previously linked to some clinical phenotypes of patients i.e., active smokers, elderly patients with long duration of asthma, presence of fixed airflow obstruction and severe symptoms [35, 42]. The limit of most of the studies is that they analyze the association of a single features with SAD, instead of doing a comprehensive evaluation of the features associated with SAD. Therefore, we have undertaken a study to identify the predictors of SAD, and were able to show the association with SAD for increased fractional exhaled nitric oxide (odds ratio [OR] 2.05; 95% CI 1.14–3.70), female sex (OR 2.27; 95% CI 1.29–4.06), smoking (OR 3.06; 95% CI 1.60–6.05), age > 50 years (OR 3.08; 95% CI 1.77–5.49), asthma-related night awakenings (OR 3.34; 95% CI 1.85–6.17), overweight with body mass index > 25 kg/m^2^ (OR 3.64; 95% CI 1.99–6.85), and exercise-induced asthma symptoms (EIA, OR 6.39; 95% CI 3.65–11.45) were independent predictors of SAD. Of note, we performed a classification tree analysis which may further help in distinguishing patients with SAD. Both the analyses concluded that EIA was the most important factor associated with the presence of SAD, followed by overweight and night awakenings due to asthma. Interestingly, the decision tree analysis showed that overweight asthmatic patients with EIA would have a 94% prevalence of SAD, and in those without EIA but with night awakenings due to asthma the prevalence of SAD would be 66% of patients [34]. We concluded that these associations may be of help in distinguishing subjects with SAD among patients with asthma, especially when IOS cannot be performed.

### IOS-defined SAD as a “treatable trait”?

Dissect the “umbrella” of airway diseases into components before planning treatment, with a focus on traits that are identifiable and treatable (i.e., treatable traits), is essential for deploy airway precision medicine in clinical practice [100, 101]. Most inhaled therapies do not sufficiently reach the small airways, and this inability to reach and treat the peripheral airways may strongly contribute to the lack of efficacy of inhaled treatments [102, 103]. Therefore, involvement of distal airways in asthma and COPD have justified research efforts to create pharmacologic treatments and technologies that can reach and target the peripheral airways, i.e., extra-fine inhaled formulations. Extra-fine formulations, with a mass median aerodynamic diameter (MMAD) of approximately 1–1.5 μm, have a higher lung deposition (50–60%) than coarse particle ICSs with an MMAD of 3–4 μm (10–20%) and then penetrate more deeply into the peripheral airways than drugs delivered via traditional inhalers [104–106]. Importantly, small-particle aerosols are not exhaled to any significantly greater level compared to large-particle aerosols, when assessed using in vivo lung deposition studies [107]. Recently, the HFA-propelled extra-fine fixed combination formulation of beclomethasone dipropionate/formoterol (BDP/F) 100/6 μg has been developed [108, 109] and represents the only extra-fine combination in both the pMDI and DPI formulations developed thus far [109]. Many real-life studies showing that the use of extrafine-particle ICS or ICS/LABA therapy is associated with a higher percentage of patients with well controlled asthma based on their Asthma Control Test and ACQ scores, compared to the use of large-particle combination treatment [110–115]. In our *real life* study [34], only 16.3% of patients with SAD were treated with inhaled extra-fine therapy compared to 60.4% of patients without SAD. Similarly, patients at BTS step two treated with inhaled extra-fine ICS demonstrated significantly reduced airway resistance compared to patients receiving standard particle size ICS at this step [116]. In a Mexican study, an extra-fine combination of ICS/LABA improved the level of asthma control in patients after 1 month of treatment, a result which is sustained after 3 months. Likewise, this improvement showed a tendency to correlate with the improvement in lung function measured by IOS [117].

On the other hand, step-up to high-dose combination treatment in uncontrolled asthma is associated with improved peripheral airway function as measured by Xrs5Hz and MBNW [63]. Effects of small-particle long-acting β-agonists on the small airways have been poorly documented. Manoharan et al. showed significant improvements in IOS but not spirometry after chronic dosing with formoterol (small-particle) compared with salmeterol (large-particle), both in association with ICS for 1–2 weeks with a 1- to 2-week washout period in between [118].

Taken altogether, extra-fine ICS particles (ICS and ICS/LABA) seem to better penetrate in distal airways, with additional clinical benefits in the treatment of asthma compared with coarse-particle treatment, contributing toward the observed better asthma control in real-life studies. These results, however, needs to be confirmed on larger and better designed studied in order to clearly demonstrate the potential of extrafine therapy on asthma control.

IOS-defined SAD may be modified by biologics, and particularly antiIL-5 monoclonal antibody mepolizumab was able to improve lung ventilation heterogeneity indexes in subjects with severe asthma after treatment [119, 120]. Antonicelli and colleagues evaluated the role of FOT in monitoring the effects of mepolizumab treatment in severe eosinophilic asthma, which correlated with both eosinophil counts and asthma control scores [121]. Controlling eosinophilic bronchitis with anti-T2 therapies improves ventilation defects, measured by inhaled gas MRI, in adults with prednisone-dependent asthma [122]. The effects of treatment with biologics on plethysmographic and IOS parameters and on ventilation heterogeneity assessed with the multiple-breath nitrogen washout and inhaled hyperpolarized 129Xe MRI will be investigated in ongoing trials [48].

### Conclusion

Despite the availability of effective therapies, a substantial proportion of asthmatics remain poorly controlled in *real life*. Given the clinical impact of SAD on asthma control, SAD should be actively searched as part of the daily management of patients with asthma.

Objective markers sensitive to both large and small airway mechanics are needed to complement the currently broadly accessible conventional spirometry. Among others, IOS is a noninvasive and effort-independent method for the detection of SAD in asthma.

Since asthma control has been extensively proved to be linked with SAD, and specifically more intimately with the IOS-defined SAD phenotype than to FEV1 per se, IOS should complement spirometry as part of the routine work up of asthma patients in a *real-lif*e clinic setting.

In clinical routine practice IOS is only rarely used, and when IOS cannot be performed, risk factors for SAD (uncontrolled asthma, exercise-induced symptoms, overweight, nocturnal symptoms due to asthma, active smoking, older age, fixed airflow obstruction and allergic asthma) should be investigated during clinical history collection. Finally, the identification of SAD during the diagnostic work up influence the treatment choice. Therefore, IOS may be of great help to better characterize SAD as “treatable trait”, leading to a more targeted asthma management and individualized patient care.

## Data Availability

Not applicable.

## Abbreviations

CT: Computed tomography
COPD: Chronic obstructive pulmonary disease
FOT: Forced oscillometry technique
IOS: Impulse oscillometry
MRI: Magnetic resonance imaging
SAD: Small airway dysfunction
